# Genome-wide siRNA screen of genes regulating the LPS-induced TNF-α response in human macrophages

**DOI:** 10.1038/sdata.2017.7

**Published:** 2017-03-01

**Authors:** Jing Sun, Samuel Katz, Bhaskar Dutta, Ze Wang, Iain D.C. Fraser

**Affiliations:** 1 Signaling Systems Unit, Laboratory of Systems Biology, National Institute of Allergy and Infectious Diseases, National Institutes of Health, Bethesda, Maryland 20892, USA; 2 Department of Veterinary Medicine, University of Cambridge, Madingley Road, Cambridge CB3 0ES, UK; 3 Bioinformatics Team, Laboratory of Systems Biology, National Institute of Allergy and Infectious Diseases, National Institutes of Health, Bethesda, Maryland 20892, USA; 4 Lymphocyte Biology Section, Laboratory of Systems Biology, National Institute of Allergy and Infectious Diseases, National Institutes of Health, Bethesda, Maryland 20892, USA

**Keywords:** Toll-like receptors, High-throughput screening, Inhibitory RNA techniques, Microarray analysis, Systems biology

## Abstract

The mammalian innate immune system senses many bacterial stimuli through the toll-like receptor (TLR) family. Activation of the TLR4 receptor by bacterial lipopolysaccharide (LPS) is the most widely studied TLR pathway due to its central role in host responses to gram-negative bacterial infection and its contribution to endotoxemia and sepsis. Here we describe a genome-wide siRNA screen to identify genes regulating the human macrophage TNF-α response to LPS. We include a secondary validation screen conducted with six independent siRNAs per gene to facilitate removal of off-target screen hits. We also provide microarray data from the same LPS-treated macrophage cells to facilitate downstream data analysis. Tertiary screening with multiple TLR ligands and a microbial extract demonstrate that novel screen hits have broad effects on the innate inflammatory response to microbial stimuli. These data provide a resource for analyzing gene function in the predominant pathway driving inflammatory cytokine expression in human macrophages.

## Background & Summary

Toll-like receptors (TLR) are a major class of pattern recognition receptors in innate immune cells, sensing a wide range of bacterial, viral, and microbial stimuli^1,2^. Downstream effectors of the activation of TLR pathways include many of the most important transcription factors involved in the eventuation of the host’s immune response^2^. TLR4, a TLR conserved in human and mouse whose activation is central to the immune response to gram-negative bacteria^3,4^, is the most widely studied TLR with established roles in clinical cases such as sepsis and endotoxemia^5,6^. Due to its critical and initiating role in the inflammatory cytokine response, the TLR pathway has been the target of screening studies to identify novel regulators from across the genome^7–11^. However, prior studies have primarily utilized mouse cells, necessitating further validation of the applicability of these studies to the regulation of the TLR4 pathway in humans. Additionally, given the intricacy of the signaling pathways shared across multiple TLR receptors, more remains to be understood of how the candidate regulators identified by these studies are shared across multiple TLRs and which are specific to the TLR4 response. To address these challenge we have conducted a genome-wide siRNA screen in the human THP-1 macrophage-like cell line, modified to express a dual luciferase reporter for an inflammatory readout and corrective for perturbation effects on cell viability^12^. The cells were stimulated by lipopolysaccharide (LPS), a TLR ligand specific to the TLR4 receptor^3,4^. Following validation by secondary screening, robust regulatory candidates were screened for their effect in response to an array of ligands specific to various TLR receptors. Our screen provides a comprehensive genome-wide study of the global regulation of the TLR4 response in a human cell line, identifying multiple novel regulators. It can also serve as a complimentary data set to genome-wide studies in mouse cell lines for comparative analysis of conserved versus species-specific regulators, and whether the pathways downstream of multiple TLR receptors share these regulators.

To achieve the genome-wide perturbation of gene expression in the primary screen, cells were transfected with a library of siRNA SMARTpools, containing 4 siRNAs targeting each of the 18,110 genes in the human genome. Following transfection with siRNA the cells were stimulated with LPS and incubated for four hours. The cells were analyzed using a dual luciferase assay where the ratio of firefly luciferase activity (driven by the human *TNF* promoter), to renilla luciferase activity (driven by the *UBC* promoter), was taken as a readout of the gene perturbation effect on the TLR4 response. Following normalization, strong hits from the primary screen were selected for secondary validation using six individual siRNA from two different sources to facilitate removal of off-target hits from the primary screen. Robust hits from the secondary screen were selected for tertiary screening where the dual luciferase reporter cells were stimulated by a diverse range of TLR ligands. Transcriptomic analysis of the reporter cell line under the same conditions of LPS stimulation was used to filter for hits clearly expressed in target cells.

Our primary screen identified 345 positive and 231 negative putative novel regulators that were not directly associated with the TLR4 signaling pathway. Analysis of these genes in a secondary screen, and filtering by transcriptomic analysis, identified 26 novel positive regulators and 13 negative regulators of LPS-induced TNF-α induction. Of the positive and negative regulators identified in the secondary screen, 24 and 8, respectively, were identified in the tertiary screen as having a regulatory role in the response to at least one additional TLR ligand. Our data set provides a comprehensive analysis of the regulation and modulation of the TLR4 response in human macrophages. Further analysis of the data can be used for the identification of novel regulatory candidates, as well as for comparative analysis of similar studies using cell lines from other species. Additionally, data from our tertiary screen utilizing diverse TLR ligands can be used for further analysis of shared and specific regulators across the TLR receptors and their intricate downstream signaling cascades.

## Methods

### Cell culture and TLR ligand stimulation

**Figure 1:**
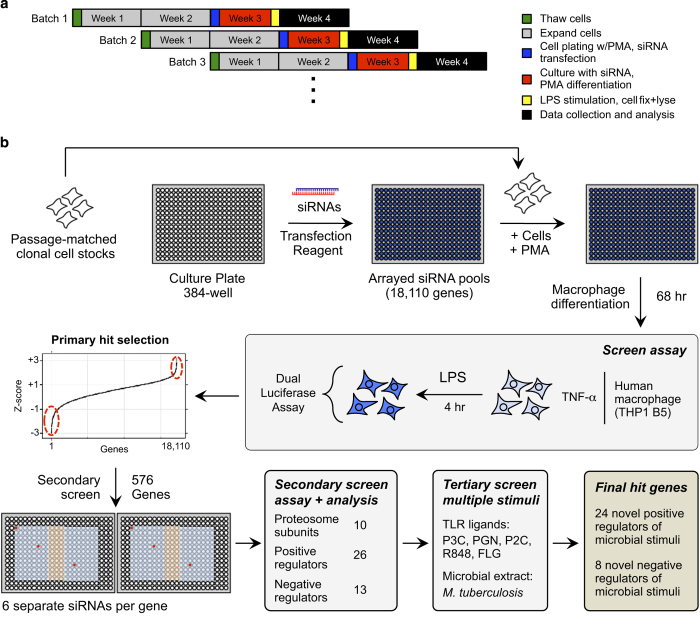
Overview of screen workflow. (**a**) Schematic of procedure to minimize cell line variation in screen. All THP1 B5 cell batches used were from a single parental low passage stock. (**b**) Overview of the procedure for primary, secondary and tertiary siRNA screens of the human macrophage TNF-α response to microbial stimuli. For the secondary screen plates, red circles show an example of siRNA locations for a single gene, blue region of plate=gene specific siRNAs and orange region=control siRNAs.

The generation of the THP1 B5 reporter cells with dual luciferase readouts for TNF-α transcriptional induction has been described previously^12^. THP1 B5 cells were maintained in RPMI, 10%FBS, 10 mM Hepes, and 2 mM glutamine. A large batch of low passage THP1 B5 cells sufficient for the entire screening process were prepared and frozen together. A new batch of cells were thawed each week throughout the screening process, and each batch were cultured for exactly 14 days prior to siRNA transfection, to ensure the same cell passage number was used for every experiment in the screen (Fig. 1a). LPS was from Alexis Biochemicals, Salmonella minnesota R595 TLRgrade, ALX-581-008-L002.

### High throughput siRNA screening

#### Overview

**Figure 2:**
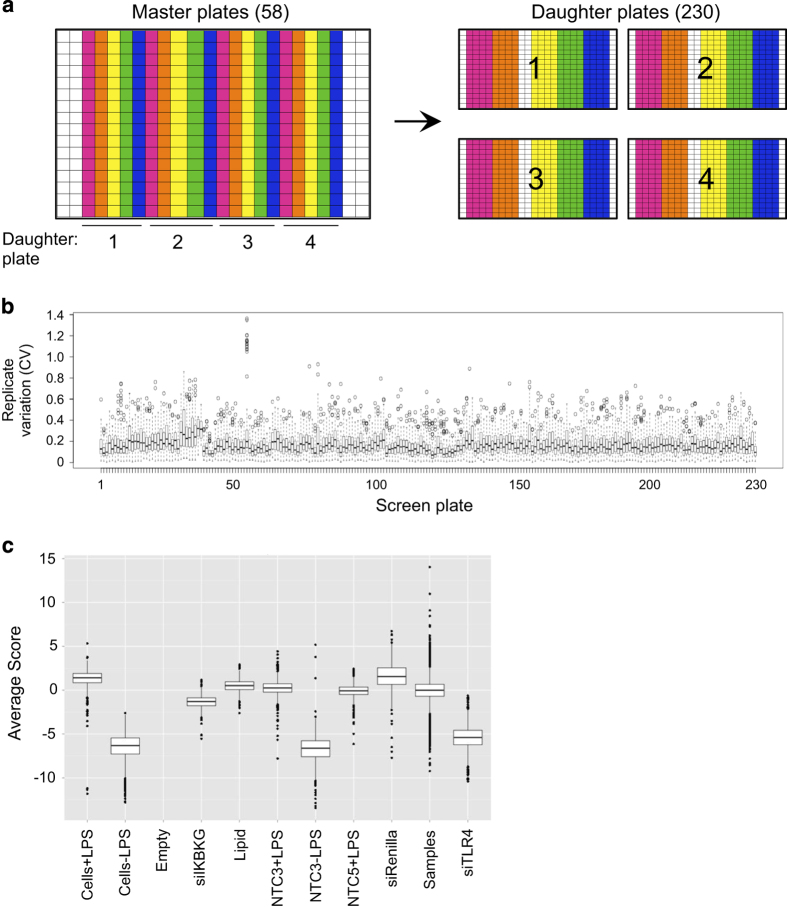
Primary screen QC metrics. (**a**) Schematic for reformatting of 58 primary screen siRNA master plates to 230 daughter assay plates containing four replicate wells of each siRNA SMARTpool. Colors indicate position of sample siRNAs, white indicates wells left open for screen controls. (**b**) Boxplots showing variation of siRNA replicates across all 230 primary screen plates. (**c**) Boxplots of negative and positive control performance in the primary screen. All conditions except siRenilla include the LPS stimulus unless otherwise indicated.

The genome-wide siRNA screen was run in 384-well format. We used the GE Dharmacon Human siGENOME SMARTpool siRNA library RefSeq27, containing a single pool of 4 siRNAs targeting each of 18,110 genes across 58 plates. For the primary screen, each library plate was reformatted to 4 assay plates (230 total screen plates) to obtain four replicate data points for each siRNA SMARTpool (Fig. 2a), as we observed better replicate correlation within rather than across plates from our dual luciferase assay in preliminary experiments (see ‘Technical Validation; Replicate correlation’). For the secondary screen, in which individual sequences from different vendors were used, three replicates of each plate were run in successive weeks. TNF-α transcriptional activation was measured by human *TNF* promoter driven firefly luciferase expression, normalized by human *UBC* promoter driven renilla luciferase expression (see Fig. 1b). Passage matched cells were used throughout the screening process to minimize cell line variability. For the primary screen, plates were prepared with siRNAs against target genes in columns 2–9 and 12–23, with controls (at least 3 wells each) in columns 10, 11, and 24, column 1 was not used. siRNA concentration throughout the primary and secondary screens was fixed at 50 nM, previously identified as optimal for the THP1 B5 line^12^. Negative controls included transfection lipid alone, non-targeting control siRNA NTC3 and NTC5. Positive controls were chosen from the canonical TLR4 pathway; *TLR4* and *IKBKG*. siRNA targeting *Renilla* luciferase was used as control for monitoring transfection efficiency. All cell plating and liquid handling steps were conducted with a Multidrop dispenser (Thermo Fisher) and EL406 plate washer/dispenser (Biotek). Luciferase activity from cell lysates was measured using the Dual-Luciferase Reporter Assay (Promega) and read with a FLUOstar Omega plate reader (BMG Labtech).

#### Screening reagents

GE Dharmacon Human siGENOME SMARTpool siRNA Library RefSeq27 (G-004675-02; G-004655-02; G-005005-02).

Custom Ambion Silencer Select siRNA Library (secondary screen)

Custom Qiagen FlexiPlate siRNA Library (secondary screen)

Negative control siRNAs (GE Dharmacon); NTC3 (D-001210-03), NTC5 (D-001210-05)

Positive control siRNAs (GE Dharmacon except *MAP3K7*); *TLR4* (M-008088-01), *IRAK1* (M-004760-03), *IKBKG* (M-003767-02), *MAP3K7* (Qiagen, S100300741), custom *Renilla* siRNA^12^.

384 well plate (Falcon, Cat # 353988)

Hiperfect transfection reagent (Qiagen, Cat # 301709 )

Opti-MEM I (Life Technologies, cat # 31985070)

Phorbol-12-myristate-13-acetate (PMA) (Sigma, Cat # P1585)

Dual-Luciferase Reporter Assay System (Promega, Cat # E1960)

Phosphate buffered saline (Gibco, Cat # 10010-023)

RPMI 1640 (Lonza, 12–167F)

Fetal bovine serum (GemCell, 100–500)

Hepes (Corning, 25-060-CL)

L-glutamine (Lonza, 17-605E)

Lipopolysaccharide (LPS) (Alexis Biochemicals, Cat # ALX-581-008-L002)

Pam3CSK4 (P3C) (InvivoGen, Cat. No. tlrl-pms).

PGN (Sigma, peptidoglycan from Staphylococcus aureus, Cat.# 77140).

R848 (InvivoGen, tlrl-r848).

Pam2CSK4 (P2C) (EMC Microcollections, Cat# L2020).

Flagellin (Invivogen, FLA-ST ultrapure, tlrl-epstfla).

Extract from BCG-attenuated *Mycobacterium Tuberculosis* (generously provided by Dr Carl Feng).

**Day 1: siRNA Reverse transfection.** All siRNAs were pre-arrayed in 384-well plates with 2 μl of a 1.25 μM master stock. A master stock containing 7.8 μl of Opti-MEM I and 0.2 μl of Hiperfect for each well was prepared, incubated for 5 min at room temperature, then added to each well. The plates were shaken for 1 min to generate a homogenous siRNA/lipid suspension. After 20 min of incubation of the siRNA/lipid mixture at room temperature, a 40 μl suspension of 5,000 cells in growth media containing 6.25 ngml^−1^ PMA was added to give a final siRNA concentration of 50 nM, and PMA concentration of 5 ngml^−1^ Cells were incubated at 37 °C/5% CO_2_ for 68 h.

**Day 4: LPS stimulation and cell lysate collection**. 10 μl of fresh complete growth media containing LPS at a concentration of 60 ngml^−1^ was added to each well to achieve a final concentration of 10 ngml^−1^ LPS, apart from control wells run with no LPS stimulation which received media alone. After 4 h of incubation, the cell was washed once with 80 μl phosphate buffer solution (PBS) and then lysed in 5 μl passive lysis buffer. Plates were stored in −30 °C and the dual luciferase assay was run within a week.

**Dual luciferase assays.** The Dual-Luciferase Reporter Assay was performed to determine both the firefly (R1) and renilla (R2) luciferase activity in the cell lysates, following the manufacturers protocol. Each screen plate was required to meet the following four criteria; 1) NTC control siRNA transfected cells showed an LPS-induced reporter assay readout increase of at least 5-fold over no LPS controls, 2) the knockdown efficiency in Renilla siRNA transfected control wells was at least 85%, 3) the reduction in the LPS-induced reporter assay readout in TLR4 siRNA transfected cells was at least 85% and 4) the Renilla luciferase reading did not reach saturation. The ubiquitin promoter driven renilla luciferase activity was used as a normalization factor to reflect the cell number and viability of the sample, and the *TNF* promoter driven firefly activity provided a measure of the TLR4 ligand induced *TNF* activation. The ratio of firefly to renilla luminescence (R1/R2) thus provided a corrected measure of the TNF transcriptional response to LPS stimulation.

### siRNA screen hit selection

#### Primary genome-wide screen

*Analysis*: We evaluated the correlation between the four replicate wells from each siRNA SMARTpool, and observed a consistently low CV across the primary screen (Fig. 2b), with an overall screen average CV of 0.145. The raw R1/R2 data was normalized on a per-plate basis to the intra-plate median. We then standardized the values for each replicate experiment using the robust z-score calculation^13^. The robust z-score for each well in the primary screen is provided in Data record 1, however for our analysis, the mean of the robust z-score from the 4 replicate wells was taken as the final score for each gene. Boxplots of the screen samples against controls are shown in Fig. 2c. Based on the scores for known regulators of the TLR4 pathway, we chose a mean score of −1.5 as the threshold for putative positive regulators of LPS-induced TNF-α activation. A robust z-score of ≥2.8 was chosen for putative negative regulators. Genes identified from the canonical TLR4 pathway were noted but were not selected for the secondary screen (see ‘Identification of known pathway regulators’ section under Technical Validation), as our goal was to identify novel regulators of the LPS response. This analysis resulted in a selected gene list of 345 putative positive regulators and 231 putative negative regulators for secondary screening.

#### Secondary screen with six independent siRNAs per gene

*Methodology*: It has been shown that siRNA screens are subject to a significant frequency of off-target effects (OTEs) driven by the seed sequence of siRNAs targeting the 3′UTR of unintended gene targets by a microRNA-like targeting mechanism^14,15^. The most reliable method to separate off-target from on-target hits in an siRNA screen is to target putative hits with alternative siRNA sequences (containing different seeds) in the secondary screen. We therefore employed an additional six siRNAs from alternate vendors (three each from Ambion and Qiagen) for each of the hit genes from the primary screen. The secondary screen siRNAs were plated in 384-well format with the outer two rows and columns left empty and three central columns (11, 12 and 13) and column 22 left open for control siRNAs (see Fig. 1b). Negative and positive controls were the same as for the primary screen, with the inclusion of TAK1 (*MAP3K7*) as an additional positive control. Also, individual siRNAs for each gene were plated in separate regions of the plate (Fig. 1b, wells highlighted red show example for one gene). The secondary screen was run in 384-well format. Three replicates of each plate were run in successive weeks, and passage matched cells (from the same parental cell stock as the primary screen) were again used to minimize cell line variability (Fig. 1a).

**Figure 3:**
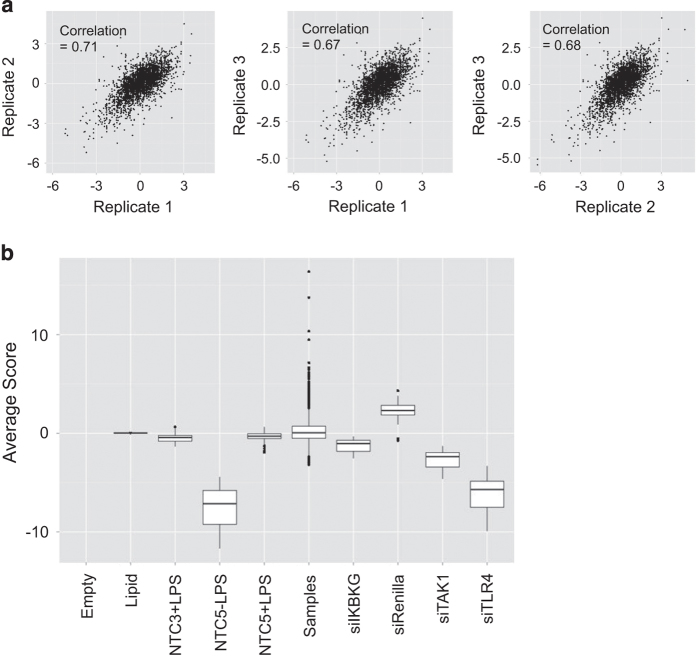
Secondary screen QC metrics. (**a**) Correlations between the three replicate experiments run for the secondary siRNA screen. (**b**) Boxplots of negative and positive control performance in the secondary screen. All conditions except siRenilla include the LPS stimulus unless otherwise indicated.

*Analysis*: For the secondary siRNA screen, data was again normalized on a per-plate basis to the intra-plate median, and we observed more normally distributed data for each replicate after log transformation (Fig. 3a). We calculated correlations of >0.65 between each pair of replicates (Fig. 3b), so the average of all three replicates was taken as the z-score for each of the 3456 siRNAs (576 genes×6 siRNA per gene) in the secondary screen. The median of the scores for the 6 siRNAs was calculated to give a single secondary screen score for each gene. All secondary screen data is included in Data record 2. We then used transcript profiling data to determine present/absent expression calls for the secondary screen genes (see Transcriptome analysis). We observed a very strong enrichment for proteasome components among the positive regulators of the LPS response, possibly due to the known requirement for proteasomal degradation of signalling proteins in the TLR4 pathway. These genes were not selected for tertiary screening. We selected 26 genes with the lowest median z-scores across the six secondary screen siRNAs, and 13 genes with elevated z-scores as novel candidate regulators of LPS-induced TNF-α induction in human macrophages (Supplementary Table 1).

#### Tertiary screening with multiple TLR ligands and microbial infection

*Methodology*: We selected the most effective secondary screen siRNA for each of the 39 candidate regulators and tested whether they also affected TNF-α transcriptional reporter induction by different TLR ligands, using the same dual luciferase assay as the primary and secondary screen. We tested the following ligands/TLR receptor targets; LPS/TLR4; Pam3CSK4 (P3C)/TLR1+2, peptidoglycan (PGN)/TLR2, R848/TLR7+8, Pam2CSK4 (P2C)/TLR2+6, Flagellin (FLG)/TLR5. We also included an extract from BCG-attenuated *Mycobacterium tuberculosis* to determine if the putative screen hits affected the macrophage response to a microbial extract.

*Analysis*: Since the tertiary screen was run on a small sample set, we evaluated the effects of the siRNAs on responses to different stimuli by the fractional effect on the *TNF*-α reporter readout compared to NTC5 negative control siRNA transfected cells. We observed that of the 26 positive regulator candidates, 24 showed a >30% signal reduction with at least one additional stimulus (Supplementary Table 2). Among the 13 negative regulator candidates, 8 showed >25% signal increase with a least one additional stimulus (Supplementary Table 2).

### Transcriptome analysis

THP1 B5 cells were treated with either 0 or 10 ngml^−1^ LPS for 4 h. Total RNA was isolated from approximately 10^6^ cells per condition using an RNAeasy Mini Kit (Qiagen). Each condition was represented by biological duplicates. cRNA amplification and labeling were performed using the Illumina TotalPrep RNA Amplification Kit (Ambion), microarray hybridization and scanning protocols followed standard Illumina protocols. Signal data was extracted from the image files with the Gene Expression module (v. 1.9.0) of the GenomeStudio software (v. 2011.1), and Log2 signal intensity and detection *P*-values were determined. Genes with *P*-value of detection of less than 0.1 were considered expressed. Genes were considered expressed in macrophages if they had a P-value of detection of less than 0.1 in at least one of the 2 conditions analyzed (+/−LPS).

## Data Records

### Data record 1

Primary screen siRNA data are available at PubChem under the accession number AID 1224830 (Data Citation 1). Raw data for the firefly (Rep1R1) and renilla (Rep1R2) luciferase readings are provided, along with the R1/R2 ratio (Rep1R1/R2). Normalized data is also included (Zscore), control wells are indicated, and samples are defined by siRNA SMARTpool ID (GE Dharmacon catalog number), Gene Symbol and EntrezID. Minimal data fields for analysis of the screen data using CARD software^16^ are included (PlateID, Well, GeneSymbol, EntrezID, siRNAID, WellAnno). The PubChem activity score indicates the phenotypic outcome for each well (0=Z-score between 1 and −1; 25=Z-score greater than 1 or less than −1; 50=Z-score greater than 2.8 or less than −1.5; 75=Z-score greater than 4 or less than −2; 100=Z-score greater than 5 or less than −2.5), and the Pubchem activity outcome notes whether the siRNA SMARTpool in a given well was considered ‘active’=2 or ‘inactive’=1. Note that all control wells were assigned a 0 activity score and an outcome of 4 by default.

### Data record 2

Secondary screen siRNA data are available at PubChem under the accession number AID 1224831 (Data Citation 2). Raw data for the three replicate experiments for firefly (Rep1-3R1) and renilla (Rep1-3R2) luciferase readings are provided, along with the R1/R2 ratio (Rep1-3R1/R2). Normalized data is also included as the replicate average for each individual siRNA (Zscore) and then the median value for the six siRNAs targeting the same gene (ZscoreGeneMedian). Control wells are indicated, and samples are defined by siRNA ID (Ambion and Qiagen catalog numbers), siRNA# (1–6 for each gene target), Gene Symbol and EntrezID. Minimal data fields for analysis of the screen data using CARD software^16^, are included (PlateID, Well, GeneSymbol, EntrezID, siRNAID, WellAnno). The PubChem activity score indicates the phenotypic outcome for each well (0=Z-score between 1 and −0.75; 25=Z-score greater than 1 or less than −0.75; 50=Z-score greater than 1.5 or less than −1; 75=Z-score greater than 2 or less than −1.5; 100=Z-score greater than 2.5 or less than −2), and the Pubchem activity outcome notes whether the single siRNA in a given well was considered ‘active’=2 or ‘inactive’=1. Note that all control wells were assigned a 0 activity score and an outcome of 4 by default.

### Data record 3

Microarray data from LPS treated and untreated THP-1 B5 cells are available at the Gene Expression Omnibus (GEO) within the data series record GSE83826 (Data Citation 3). Three replicate data files are provided for each condition, containing Log2 signal intensity and detection *P*-values for each Illumina probe ID.

## Technical Validation

### Plate uniformity

Upon establishing the dual reporter screen assay in the PMA differentiated THP1 B5 macrophages, we initially assessed the plate uniformity of the assay in 384-well format to determine if we could use the entire plate and avoid edge effects and other positional biases. Following the guidelines established by the NCATS screening facility^17^, intra-plate uniformity was tested. The same assay as used in the siRNA screen was run in 384-well replicate plates using negative control siRNA NTC5. Each plate was treated with 3 different doses of LPS (High, Medium and Low (H, M, L)) to give increasing levels of activation. The H, M, L wells were arranged alternately within one plate (Repeating pattern of 2 columns of H, 2 columns of M, 2 columns of L) and staggered among the 3 replicate plates (one starting HML, one MLH, one LHM). To calculate the uniformity, wells from each LPS dose were divided into 4 groups, and the mean of each group readout was calculated. The maximal difference between a pair of group means was then divided by the mean of all the 4 groups receiving the same dose of LPS to obtain the parameter reflecting the uniformity of the assay. A difference of less than 20% between all groups of a given dose is considered as no edge effect. As shown in Table 1, no edge effect was observed in the assay, permitting the use of entire 384-well plates in the primary screen.

### Replicate correlation

In preliminary experiments testing the reproducibility of the dual luciferase assay, we observed lower CVs for replicates run within plates rather than across plates (18.61 versus 23.03%). This led us to the plate reformatting strategy for the primary screen, where four replicate wells were run for each siRNA SMARTpool (Fig. 2a), and these replicates showed consistently low CVs across the primary screen (Fig. 2b), with an overall screen average CV of 0.145. Throughout the secondary screen, three replicates of each plate were run in successive weeks, and correlations exceeding 0.6 for all pairwise replicate comparisons are shown in Fig. 3a.

### siRNA transfection efficiency

In developing the THP1 B5 reporter clone, we established a method for optimizing siRNA transfection efficiency whereby we targeted the constitutively expressed Renilla reporter with a pool of potent siRNAs against the Renilla mRNA^12^. We included 3 siRenilla and 3 NTC5 control wells on every screening plate that were not treated with LPS to assess siRNA transfection efficiency. Average Renilla knockdown levels exceeded 95% throughout the primary and secondary screens and are shown in Table 2.

### Control performance

We included multiple positive controls on every screening plate targeting known components in the LPS/TLR4 pathway, including the TLR4 receptor, the TLR pathway kinase TAK1 (*MAP3K7*), and the regulatory component of the NF-κB pathway kinase complex NEMO (*IKBKG*). Boxplots from the primary and secondary screens (Figs 2c and 3b), show varying levels of pathway perturbation with these controls, which we used as a metric for assessing thresholds for likely hits in the screen.

### Identification of known pathway regulators

The primary genome-wide screen identified numerous known components of the TLR4 signaling pathway among the top hits including components of the receptor complex *TLR4* (−2.3), *LY96* (−1.7), and *CD14* (−1.5), and the TIR domain receptor binding adaptors *MYD88* (−1.84) and *TIRAP* (−1.94). Top hits also included the key TNF-α inducing transcription factors *SPI1* (−2.3), *ATF7* (−2.2), *JUN* (−2.1), *SP1* (−1.88), *ELK4* (−1.79) and *EGR1* (−1.72). We chose not to include canonical pathway regulators in the secondary screen described here, as we have previously published a rigorous RNAi screen of over 100 genes from the canonical TLR pathways, using multiple siRNAs per gene and stimulating macrophages with a panel of up to six TLR ligands^11^.

## Usage Notes

### Data files for screen analysis using CARD software

We recently described a software package for comprehensive analysis of RNAi screen data, which combines both existing and novel algorithms for data pre-processing, reducing false positive hits through gene expression and off-target filtering, implementing network/pathway enrichment of high-confidence hits and predicting active miRNAs^16^. The data we describe in Data records 1 and 2 (Data Citations 1 and 2) include all the required fields to permit analysis of the screen data in CARD (PlateID, Well, GeneSymbol, EntrezID, siRNAID, WellAnno). Instructions for uploading and analyzing the data in CARD have been previously described^16^.

## Additional Information

**How to cite this article**: Sun, J. *et al.* Genome-wide siRNA screen of genes regulating the LPS-induced TNF-α response in human macrophages. *Sci. Data* 4:170007 doi: 10.1038/sdata.2017.7 (2017).

**Publisher**’**s note**: Springer Nature remains neutral with regard to jurisdictional claims in published maps and institutional affiliations.

## Supplementary Material



Supplementary Table 1

Supplementary Table 2

## Figures and Tables

**Table 1:** Assessment of the plate uniformity of the screening assay.

	**High dose (LPS: 10 ngml^−1^)**	**Middle dose (LPS: 1 ngml^−1^)**	**Low dose (LPS: 0 ngml^−1^)**
Plate set 1 variation	18.3%	5.4%	16.2%
Plate set 2 variation	6.5%	11.2%	6.2%
Plate set 3 variation	18.4%	8.9%	10.0%

**Table 2:** Knock down efficiency of Renilla luciferase activity by Renilla siRNA in primary and secondary screens.

	**Mean**	**s.d.**
Primary screen	96.4%	1.7%
Secondary screen	97.4%	2.6%
